# Sesame Lignans Suppress Age-Related Cognitive Decline in Senescence-Accelerated Mice

**DOI:** 10.3390/nu11071582

**Published:** 2019-07-12

**Authors:** Satomi Shimoyoshi, Daisuke Takemoto, Yoshiko Ono, Yoshinori Kitagawa, Hiroshi Shibata, Susumu Tomono, Keiko Unno, Keiji Wakabayashi

**Affiliations:** 1 Institute for Health Care Science, Suntory Wellness Limited, Kyoto 619-0284, Japan; 2 Graduate Division of Nutritional and Environmental Sciences, University of Shizuoka, Shizuoka 422-8526, Japan; 3 Department of Microbiology and Immunology, Aichi Medical University, Aichi 480-1195, Japan; 4 School of Pharmaceutical Sciences, University of Shizuoka, Shizuoka 422-8526, Japan

**Keywords:** sesame lignans, sesamin, episesamin, cognitive decline, depression, SAMP10, senescence-accelerated mouse, reactive carbonyl

## Abstract

Sesame lignans, which are biologically active compounds present in sesame seeds and oil, are known to have neuroprotective effects in several models of brain dysfunction. However, the effects of sesame lignans on age-related brain dysfunction are not clear and were thus investigated in the present study using a senescence-accelerated mouse (SAMP10). Two-month-old male SAMP10 mice were administrated a basal diet with 0% or 0.05% sesame lignans for two months, or with 0%, 0.02%, or 0.05% sesame lignans for 10 months and subjected to step-through passive avoidance tasks and forced swim tests. Reactive carbonyl species (RCs) were evaluated as markers of oxidative stress using a recently developed comprehensive analytical method. Both learning time in passive avoidance tasks and immobile time in forced swim tests became longer with aging (*p* < 0.05). However, the administration of sesame lignans significantly ameliorated age-related effects in both tests (*p* < 0.05). Age-related increases in RCs such as 4-hydroxy-2-nonenal in the cerebral cortex and liver were reduced in mice fed sesame lignans. These results suggest that sesame lignans can prevent age-related brain dysfunction via anti-oxidative activity.

## 1. Introduction

Aging is an inevitable biological process characterized by a gradual decline in physiological function that can lead to various age-related disorders. Cognitive decline including impaired memory and learning ability is a well-known age-related disorder and might, as it progresses, lead to dementia. Dementia is among the major reasons that aging people require nursing care and results in seriously impaired quality of life. Thus, cognitive decline in the elderly, and the high social and economic burdens associated with it, have become an important public health concern [1].

A number of studies have suggested that oxidative damage accumulates in the brains of patients with Alzheimer’s disease and dementia [2], and hence, oxidative stress is considered one of the main causes of age-related cognitive decline. Oxidative stress causes damage to cellular components including polyunsaturated fatty acids (PUFAs), DNA, and proteins, leading to the onset or acceleration of degenerative disorders [3]. Human brains produce excessive free radicals because they consume approximately 20% of total body oxygen despite comprising less than 2% of total body weight. In addition, the brain is rich in easily oxidizable PUFAs and transition metals, which are key players in oxidation that promote the formation of oxygen free radicals. Reactive carbonyl species (RCs) are produced by lipid peroxidation and contain highly reactive carbonyl groups. RCs such as 4-hydroxy-2-nonenal (HNE) react directly with amino acids, nucleic acids, and phospholipids, impairing the structure and function of these molecules. RCs are known to increase the risk of cardiovascular disease, chronic diseases [4], non-alcoholic fatty liver disease [5], and cancer [6]. We previously developed a novel comprehensive analytical method to profile lipophilic RCs in biological samples using liquid chromatography/electrospray ionization tandem mass spectrometry (LC/ESI-MS/MS) with selected reaction monitoring [7]. Using this method, we showed that RCs are present in colorectal cancer cells and the gastric mucosa of mice with colorectal cancer [8,9]. It has been reported that the major RCs such as HNE [10] and 4-hydroxy-2-hexenal (HHE) [11] are correlated with cognitive decline. However, the manner in which RCs in the brain change with aging and the relationship between age-related brain function and the content of these molecules in the brain have not been clarified.

The senescence-accelerated mouse (SAM), a murine model of accelerated senescence, was established by Takeda et al. [12]. Among SAM strains, SAMP10 shows impaired learning at the age of 10–12 months with age-related brain atrophy [13,14] and behavioral depression [15,16], which are thought to be caused by reactive oxygen species (ROS). We have previously shown that the administration of catechin and β-cryptoxanthin suppresses age-related cognitive decline and the increase in 8-oxodG in the cerebrum, due to the anti-oxidative activity [17,18]. The SAMP10 strain might thus be a useful model for age-related dementia and depression, as morphological alterations in its cortical neurons are similar to those in the aging human brain [19]. In addition, those changes start to appear at approximately 8–10 months of age in SAMP10 mice, which is earlier than that in other strains such as C57BL/6 that exhibit normal aging [20].

Sesamin is a natural constituent of sesame (*Sesamum indicum*) seed and oil. Almost half of sesamin is epimerized during the refining of non-roasted sesame seed oil. It has also been reported that sesame lignans, which contain sesamin and episesamin, have a variety of physiological effects including anti-oxidative [21,22], hypocholesterolemic [23], anti-hypertensive [24], liver protective [25], and neuroprotective [26,27]. We have previously demonstrated that the metabolites of sesamin and episesamin; SC1 ((7α,7′α,8α,8′α)-3,4-dihydroxy-3′,4′-methylenedioxy-7,9′:7′,9-diepoxylignane), EC1 ((7α,7′β,8α,8′α)-3,4-dihydroxy-3′,4′-methylenedioxy-7,9′:7′,9-diepoxylignane), or ((7α,7′β,8α,8′α)-3,4-methylenedioxy-3′,4′-dihydroxy-7,9′:7′,9-diepoxylignane); produced by P-450 have potent antioxidative effects [21,28]. SC1 and EC1 are also capable of activating Nrf2/ARE signaling and are protective for rat pheochromocytoma PC12 cells, widely used as a model for neural differentiation [29]. In vivo, sesamin was also found to exhibit neuroprotective effects in a rat stroke model by suppressing microglia activation, MAP kinase activation, and inflammation [26]. Further, Fujikawa et al. [27] reported that sesame lignans have a positive effect on behavioral dysfunction in a rotenone-induced parkinsonian model. However, the effect of sesame lignans on age-related brain dysfunction remains unclear.

Here, we examined whether sesame lignans affect age-related cognitive decline in SAMP10 mice. We also used a comprehensive analytical method to determine age-related changes in brain RCs in these mice and characterized the effects of sesame lignans.

## 2. Materials and Methods

### 2.1. Materials

Sesame lignans (comprising approximately equivalent amounts of sesamin and episesamin) were obtained from TAKEMOTO OIL & FAT Co., Ltd. (Aichi, Japan). The purity of sesame lignans, analyzed by HPLC, was 99.8% (sesamin: 47.1%, episesamin: 52.7%). Dansyl hydrazine (DH) was obtained from Invitrogen (Carlsbad, CA, USA). p-Toluenesulfonic acid (p-TsOH) and RCs including propanal, butanal, heptanal, pentanal, 2-hexenal, hexanal, 2-heptenal, octanal, 2-nonenal, nonanal, decanal, undecanal, dodecanal, and tridecanal were purchased from Sigma-Aldrich (St. Louis, MO, USA). 4,5-Epoxy-2-decenal (EDE), HHE, and HNE were obtained from Cayman Chemical Company (Ann Arbor, MI, USA). Crotonaldehyde, 2,4-nonadienal, 2,4-decadienal, pentadecanal, hexadecanal, heptadecanal, octadecanal, and tetradecanal were obtained from Tokyo Chemical Industry (Tokyo, Japan). p-Benzyloxybenzaldehyde (p-BOBA), formaldehyde, acetaldehyde, acrolein, 2,4-hexadienal, 2,4-heptadienal, 2-octenal, 2-undecenal, 8,11,14-heptadecatrienal, 8,11-heptadecadienal, 8-heptadecenal, and all the other chemicals were obtained from Wako Pure Chemical Industries (Osaka, Japan). Secosterols-A and -B were synthesized as previously described [30], and their purities were verified by TLC and 1H NMR analyses. Briefly, secosterol-A (289 mg, 30% from cholesterol) was obtained by cleaving the double bond of cholesterol (1 g) with ozone. Secosterol-B (158 mg, 63% from secosterol-A) was obtained from secosterol-A (250 mg) via aldolization. An internal standard (IS; p-BOBA, 10 μM) and stock solutions of the RCs were prepared separately in acetonitrile and stored at −20 °C prior to use.

### 2.2. Animals and Diets

Male SAMP10/TaSlc (SAMP10) mice were purchased from Japan SLC (Shizuoka, Japan) and maintained under conventional conditions in a temperature- and humidity-controlled room with a 12-h light/dark cycle (temperature, 23 ± 1 °C; relative humidity, 55% ± 5%; light period, 08:00–20:00). Six mice were housed per cage. Mice were fed a basal diet (AIN-76; Oriental Yeast Co., Ltd., Tokyo, Japan) ad libitum until experiment inception. Mice had free access to tap water during the experiment. The experimental diets were mixed by replacing an equal amount of corn starch with 0.02% or 0.05% sesame lignans in a basic diet (AIN-76) and pelletized by heating and pressing (Oriental Yeast Co., Ltd., Tokyo, Japan). One hundred and twelve two-month-old mice were divided into five groups (*n* = 16–28) as follows: (1) Young control, fed basal diet (YC), (2) young, fed basal diet supplemented with 0.05% sesame lignans (YS0.05), (3) old control, fed basal diet (OC), (4) old, fed basal diet supplemented with 0.02% sesame lignans (OS0.02), and (5) old, fed diet supplemented with 0.05% sesame lignans (OS0.05). Young groups and old groups were fed specific diets for two or 10 months, respectively. Body weight was measured monthly to check the condition of the animals. We carried out the experiments three times. Blood was collected from the cervical vein under anesthesia in the young groups at four months of age and in the old groups at 12 months of age. Brains and livers were removed immediately after blood collection and rinsed with ice-cold saline. After whole brains were weighed, the cerebral cortex was isolated. Each part of the tissue was stored at −80 °C until processing. To avoid technical differences arising from separation, brains of all mice were dissected by a single, expert operator. A series of studies were conducted from May 2012 to December 2017. All protocols for animal procedures were approved by the University of Shizuoka Laboratory Animal Care Advisory Committee (approval No. 136068) and the Ethics Committee of Animal Experiment of Suntory in accordance with the Internal Regulations on Animal Experiments at the University of Shizuoka and Suntory Holdings Limited, which are based on the Law for the Humane Treatment and Management of Animals (Law No. 105, 1 October 1973).

### 2.3. Step-Through Passive Avoidance Task

A step-through passive avoidance task was conducted with a step-through test system and shock generator scrambler (SGS-003, Muromachi Kikai Co., Ltd., Tokyo, Japan). The task was carried out using three-month-old mice (young groups) and 11-month-old mice (old groups) as described previously [31]. In brief, a mouse was first placed in the light room, and 1 min later the door between the dark room and the light room was opened. When the mouse entered the dark room from the light room, the door was closed, and a slight electric foot-shock (50 μA) was given for 1 s. Then, the mouse was removed from the dark room and put back in the light room. One minute later, the door was opened, and an electric shock was delivered when the mouse moved to the dark room. If the mouse remained in the light room for 300 s, the acquisition of the avoidance response was judged as successful. The trial was repeated up to five times until the mouse remained in the light room for 300 s. The time spent in the light room in each 300-s trial was recorded and then deducted from 300 s. The calculated time of each trial, up to five times, was summed as the time needed for learning and the total time was expressed as “learning time”.

### 2.4. Forced Swim Test

Immobile time in a forced swim test was assessed as an indicator of depression-like behavior [32], and performed only in the third experiment. The forced swim test was carried out with a cylinder (diameter; 17 cm) filled with water (10 cm depth, 25 °C). Mice were placed in the cylinder for 300 s the day after performing the step-through passive avoidance task. Mice initially moved vigorously when placed into water and then became immobile. The time spent floating during the trial was summed and the total was expressed as “immobile time”.

### 2.5. Measurement of Reactive Carbonyl Species

Five mice were randomly selected from each group for the measurement of RCs. RCs were analyzed by the IS method as described by Tomono et al. [7]. The p-BOBA used in IS was adopted because both the molecular weight and elution time are intermediate; in addition, many RCs including p-BOBA elute with acetonitrile and good calibration curves have been created for plasma and tissues homogenates.

Briefly, frozen mouse cerebral cortex and liver samples stored −80 °C were thawed to 0 °C on ice. After thawing, tissues were homogenized with 50 mM sodium phosphate buffer (pH 7.4) containing 20 μM dibutylhydroxytoluene and 0.5 mM ethylenediaminetetraacetic acid to make 20 mg wet tissue per 200 μL solution. The tissue homogenates were mixed with 2 μL of 10 μM IS (p-BOBA) and 400 μL of chloroform/methanol (2:1, *v*/*v*). The mixture was agitated vigorously for 1 min and centrifuged at 21,500× *g* for 10 min to collect the organic phase. The residue and aqueous phase were then mixed with 400 μL of chloroform/methanol (2:1, *v*/*v*), and was centrifuged at 21,500× *g* for 10 min to separate the organic phase. The combined organic phases were mixed with 100 μL of acetonitrile containing 50 μg of DH and 10 μg of p-TsOH, and the mixtures were incubated for 4 h at room temperature under the dark condition. The preparations were then evaporated to dry *in vacuo* to yield corresponding derivatized residues that were dissolved individually in 200 μL of acetonitrile.

Aliquots (5 μL) of samples were analyzed by LC/ESI-MS/MS (HPLC: Agilent 1200 (Agilent Technologies, Santa Clara, CA, USA), MS: G6410B Triple Quadrupole (Agilent Technologies)), using a TSK-gel Super-Octyl column (2.3 µm, 100 mm × 2.0 mm, TOSOH, Tokyo, Japan). An Agilent G6410B triple quadrupole tandem mass spectrometer equipped with an electrospray ionization device running in the positive ion mode was used. RC-DH derivatives were detected using the selected reaction monitoring (SRM) mode. The mobile phases used for the LC/ESI-MS/MS analyses were as follows: Solvent A, consisting of a 0.1% (*v*/*v*) solution of formic acid in water, and solvent B consisting of a 0.1% (*v*/*v*) solution of formic acid in acetonitrile. The DH derivatives were eluted from the column using a linear gradient, which started with 80% solvent A and 20% solvent B, and progressed to 100% solvent B over a period of 10 min. The system was then eluted with 100% solvent B for 10 min before being returned to the initial conditions over a period of 10 min, to allow for equilibration of the column. The system was operated at a constant flow rate of 0.2 mL/min for all analyses. A total of 675 SRM transitions were monitored for each DH-derivatized sample, with the transitions ranging from *m/z* 275 → 236.1 to 949 → 236.1. The ion transitions monitored for DH derivatives of IS (p-BOBA) were *m/z* 460.1 → 236.1 (t_R_: 11.38 min).

To prepare the calibration curve, we used tissues from an untreated control mouse. Tissues were homogenized and divided into tubes, equivalent to 20-mg tissue weights. Calibration curves were conducted, which involved the addition of IS (p-BOBA) and several different RCs as standard mixtures (Tables S1 and S2) to the mouse tissues. A fixed amount of IS (20 pmol, 2 μL) and each amount of RC (i.e., 0.1, 0.2, 0.5, 1, 2, 5, 10, 20, 50, and 100 pmol, 10 μL) was spiked into the homogenized mouse tissues (20 mg, 200 μL), and the resulting tissue mixtures were extracted, derivatized, and subjected to LC/ESI-MS/MS analysis. Calibration curves were constructed by plotting the peak area ratios (i.e., (total RCs–endogenous RCs)/IS). All IS ratios were obtained by subtracting the solvent-derived artifact peak as a blank. The extraction efficiency of p-BOBA was 99.4% ± 4.8%, and the relative standard deviation (R.S.D.) was 4.9%.

### 2.6. Statistical Analysis

Results were expressed as means ± SEM. Statistical analyses were performed using a one-way ANOVA followed by a Tukey–Kramer post hoc analysis. The survival rates for each group were estimated using the Kaplan–Meier method and differences in survival among the groups were tested using log-rank tests with Bonferroni correction. Statistical analyses were performed using IBM SPSS statistics 25 software (IBM, Armonk, NY, USA). *p* values less than 0.05 were regarded as statistically significant.

## 3. Results

### 3.1. Body and Brain Weights, and Survival Rates

**Figure 1:**
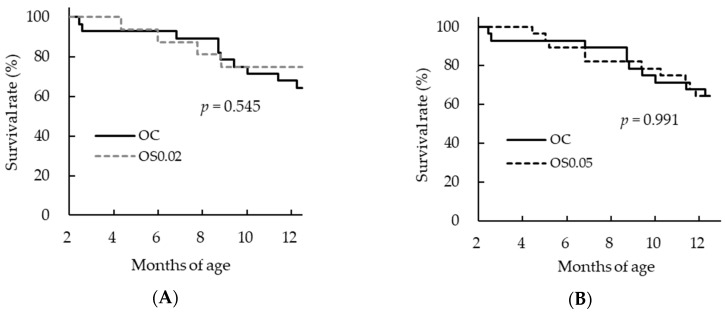
Survival curves in old mice. (**A**) Old control, fed basal diet (OC) vs. old, fed basal diet supplemented with 0.02% sesame lignans (OS0.02), (**B**) OC vs. old, fed diet supplemented with 0.05% sesame lignans (OS0.05). The Survival curves for each group were estimated using the Kaplan–Meier method and differences in survival among the groups were tested using log rank tests with Bonferroni correction. *p* values less than 0.025 were regarded as statistically significant. There were no significant differences among the groups of aged mice.

Body and brain weights were determined as general indicators of development. Body weight in the OC group at 12 months of age did not differ from that in the YC group at four months of age but was significantly lower than the body weight in the OS0.05 group (Table 1). No significant differences in brain weight were observed (Table 1). Intake of sesame lignans had no effect on survival rates in any groups (Table 1 and Figure 1).

### 3.2. Step-Through Passive Avoidance Task

**Figure 2:**
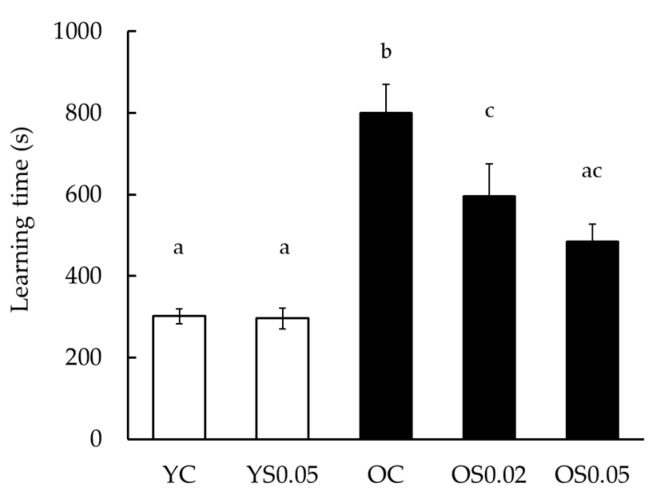
The effect of sesame lignans on passive avoidance task in young and old mice. Values are the mean ± SEM. Statistical analyses were performed using a one-way ANOVA followed by a Tukey–Kramer post hoc analysis. Different letters indicate significant differences (*p* < 0.05). Experimental groups are indicated as: Young control, fed basal diet (YC, *n* = 19), young, fed basal diet supplemented with 0.05% sesame lignans (YS0.05, *n* = 17), old control, fed basal diet (OC, *n* = 19), old, fed basal diet supplemented with 0.02% sesame lignans (OS0.02, *n* = 12), and old, fed diet supplemented with 0.05% sesame lignans (OS0.05, *n* = 21). Mice with abnormal motor function (one mouse in YS0.05, three mice in OC, and one mouse in OS0.05 groups) were excluded from analysis. The time spent in the light room in each 300-s trial was recorded and then deducted from 300 s. The calculated time of each trial, up to five times, was summed as the time needed for learning and the total time was expressed as “learning time”.

A step-through passive avoidance task was carried out to measure memory acquisition of mice in different treatment groups. The total time spent in a light room in the trials indicates learning time. The learning time of the step-through passive avoidance task was significantly longer in the OC group than in the YC group (799.6 and 301.4 s, respectively, *p* < 0.05) and the learning times in the OS0.02 and OS0.05 groups were significantly shorter than those in the OC groups (596.7 and 484.4 s, respectively, *p* < 0.05). There was no apparent difference between the YC and YS0.05 groups (Figure 2).

### 3.3. Forced Swim Test

**Figure 3:**
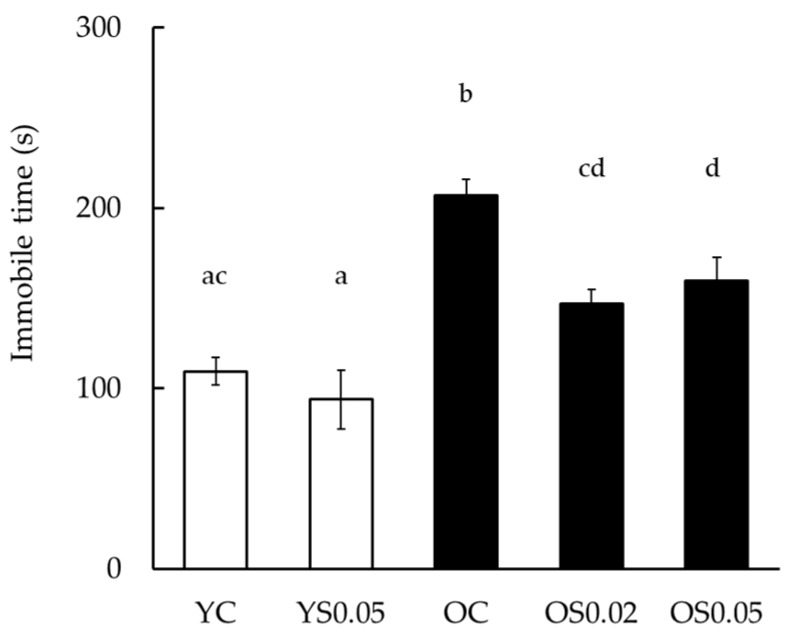
The effects of sesame lignans on a forced swim test in young and old mice. Values are the mean ± SEM. Statistical analyses were performed using a one-way ANOVA followed by a Tukey–Kramer post hoc analysis. Different letters indicate significant differences (*p* < 0.05). Experimental groups are indicated as: Young control, fed basal diet (YC, *n* = 8), young, fed basal diet supplemented with 0.05% sesame lignans (YS0.05, *n* = 7), old control, fed basal diet (OC, *n* = 9), old, fed basal diet supplemented with 0.02% sesame lignans (OS0.02, *n* = 12), and old, fed diet supplemented with 0.05% sesame lignans (OS0.05, *n* = 11).

To identify the effect of sesame lignans on depressive-like behavior, a forced swim test was conducted. In these tests, the immobile time become longer if the mouse was depressed. The immobile time was significantly longer in the OC group than in the YC group, and significantly less in the OS0.02 and OS0.05 groups than in the OC group (Figure 3). There was no significant difference between the YC and YS0.05 groups.

### 3.4. Reactive Carbonyl Species

**Figure 4:**
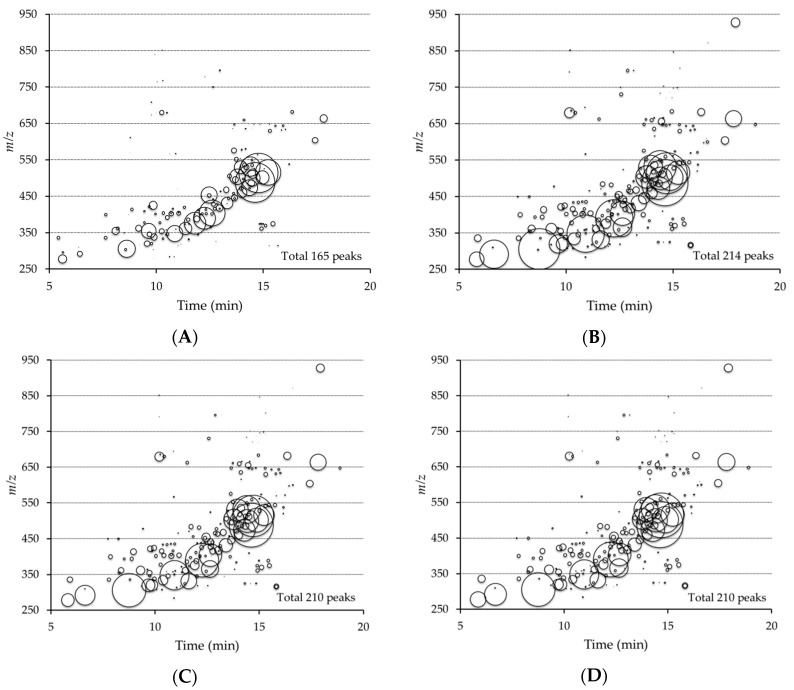
Reactive carbonyl species (RC) maps of cerebral cortices obtained from SAMP10 mice in the (**A**) YC, (**B**) OC, (**C**) OS0.02, and (**D**) OS0.05 groups. Experimental groups are indicated as: Young control, fed basal diet (YC), old control, fed basal diet (OC), old, fed basal diet supplemented with 0.02% sesame lignans (OS0.02), and old, fed diet supplemented with 0.05% sesame lignans (OS0.05). The area of each circle represents the relative intensity of the RCs compared to an internal standard. RC maps are presented as averages of each map (*n* = 5).

**Figure 5:**
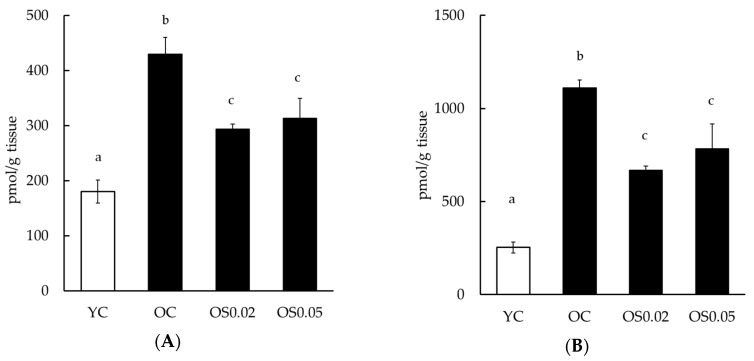
4-hydroxy-2-nonenal (HNE) (**A**) and 4-hydroxy-2-hexenal (HHE) (**B**) in cerebral cortices obtained from mice in the YC, OC, OS0.02, and OS0.05 groups (*n* = 5). Experimental groups are indicated as: Young control, fed basal diet (YC), old control, fed basal diet (OC), old, fed basal diet supplemented with 0.02% sesame lignans (OS0.02), and old, fed diet supplemented with 0.05% sesame lignans (OS0.05). Statistical analyses were performed using a one-way ANOVA followed by a Tukey–Kramer post hoc analysis. Different letters indicate significant differences (*p* < 0.05). The retention times for HNE and HHE were 10.42 min and 8.36 min, and the *m/z* information for HNE and HHE were *m/z* 362 and *m/z* 404, respectively.

The level of RCs, which are markers of oxidative stress, were comprehensively measured in the cerebral cortex and liver to elucidate the mechanisms underlying observed behavioral differences in the YC, OC, OS0.02, and OS0.05 groups. Corresponding RC maps, wherein all of the free RCs detected in the cerebral cortex samples were plotted as circles as an intersection of their retention times (horizontal axis) and *m/z* values (vertical axis), were drawn (Figure 4). The area of each circle represents the intensities of peaks for the detected RCs relative to an internal standard. The cerebral cortex obtained from the YC group contained 165 detected RCs, whereas 214 peaks were detected in the cortex obtained from the OC group. This indicates that the number of RCs in cerebral cortices increased with age. Majority of the circle areas (189 of 214) were also enlarged in the OC group compared to those in the YC group. Cortices from both the OS0.02 and OS0.05 groups were found to contain 210 peaks. However, 183 and 140 of these 210 peaks had diminished circle areas in the OS0.02 and OS0.05 groups compared to those in the OC group, respectively. The levels of major RCs related to brain function such as HNE (t_R_: 10.7 min, *m/z* 404) and HHE (t_R_: 8.7 min, *m/z* 362) are shown in Figure 5. HNE and HHE contents in the cerebral cortex were significantly higher in the OC group than in the YC group, and significantly lower in the OS0.02 and OS0.05 groups than in the OC group. The identities of 36 RCs, determined by a comparison of their retention times and *m/z* values with those of the authentic RCs, are listed in Table S1. Several RCs including butanal, 2-hexenal, hexanal, 2-heptenal, and 2,4-decadienal in the cerebral cortex were significantly higher in the OC group than in the YC group and were significantly lower in the OS0.02 and OS0.05 groups than in the OC group.

The levels of RCs detected in the liver are shown in Figure S1 and Table S2. The livers of the YC and OC groups contained 181 and 227 detected peaks, respectively, indicating that the level of RCs in the liver increases with age. The OS0.02 and OS0.05 groups contained 223 and 222 detected peaks, respectively. Several RCs including hexanal, HHE, 2,4-decadienal, HNE, hexadecanal, and octadecanal were significantly higher in the livers of mice from the OC group than in those from the YC group, and significantly lower in those from the OS0.02 and OS0.05 groups compared to those in the OC group.

## 4. Discussion

Oxidative stress is thought to be one of the main factors contributing to the onset and acceleration of brain dysfunction [33,34]. The induction of ROS generation in the brain can result in neuronal dysfunction and lead to cognitive decline and behavioral depression [35,36]. In the present study we showed that sesame lignans suppressed age-related cognitive decline and behavioral depression in SAMP10, which is a model strain that exhibits a high degree of oxidative stress in the brain due to an accumulation of ROS. Comprehensive analyses, conducted using a methodology we have recently developed [7], of RCs in SAMP10 cerebral cortices found both a greater diversity and higher concentrations of several RCs in older mice. This result is in accordance with previous data showing that levels of HNE and HHE are increased in aged brains [33]. Our comprehensive analysis revealed that sesame lignan intake suppresses the incremental increase in RCs in the aged brain. Previous studies showed that sesamin/episesamin reduce neuronal damage induced by neurotoxic chemicals through the suppression of oxidative stress, and that sesamin suppresses the decline in dopamine neurons with aging in superoxide dismutase-1 depleted *Drosophila* [37]. Furthermore, continued intake of sesamin and astaxanthin has also been suggested to improve cognitive function in humans [38]. Our current results are in good agreement with these previous studies.

Intracellular reactive oxidative stress accelerates lipid peroxidation and subsequently results in the formation of carbonyl compounds including aldehydes and ketones. RCs are considered the reactive compounds that can produce ROS in the cortex, and among them, α, β-unsaturated aldehydes, in particular, show high reactivity. Further, cysteine, lysine, and histidine residues form Michael adducts with α, β-unsaturated aldehydes, which might cause morphological and functional changes in the brain [10,39]. This is the first study to comprehensively analyze RCs in SAMP10 mice; we determined that the levels of RCs in the cerebral cortex of these animals increased with age and we also elucidated the number of RC peaks. Accordingly, learning time in a step-through passive avoidance task for 11-month-old SAMP10 mice, which was associated with a greater abundance and diversity of RCs in the cerebral cortex (Figure 4 and Figure 5), was significantly longer than that for YC mice (Figure 2). Hence, although no clear reduction in brain weight was observed with age, functional decline in the OC group, as compared to that in the YC group, was verified. Furthermore, levels of most RCs detected in this study also increased with age in the liver. These results indicate that SAMP10 is a good model to study human aging because aging is accelerated by excessive oxidative stress, which increases with age.

HNE and HHE comprise α, β-unsaturated aldehydes and have been suggested to be deeply involved in the onset and progression of cardiovascular and neurodegenerative diseases. For example, when present at high concentrations, HNE, which is one of the most highly reactive RCs, can directly bind to electron transport chain components and, subsequently to mitochondria, thereby causing electron leakage and generating increased levels of ROS [40,41]. In addition, HNE might increase mitochondrial ROS production and induce mitochondrial dysfunction indirectly by inhibiting ion motive ATPase or glucose transport [41,42]. Inhibition of either ATPase activity or glucose transport increases neuron vulnerability to oxidative injury [42,43,44]. HNE has been detected in the serum and brain tissue of patients and model animals of Alzheimer’s disease and Parkinson’s disease [10] and increases with age [45]. Williams et al. [46] reported that elevated HNE content in brains is correlated with the progression of cognitive decline. Similarly, HHE is also elevated in the central nervous system of patients with neurodegenerative diseases such as Alzheimer’s disease, Parkinson’s disease, and amyotrophic lateral sclerosis [47]. HHE is also known to induce ROS and is neurotoxic [48]. Although the HHE concentration in cerebral cortices in the current study was lower than that reported in previous studies, chronic exposure to HHE might induce ROS and oxidative damage. These results indicated that HNE and HHE might play an essential role in age-related cognitive decline in SAMP10 mice.

In the present study, we demonstrated that the intake of sesame lignans decreases age-related increases in RCs. It is thought that these effects result in the suppression of neuronal cell death and brain function deterioration with aging. Keller et al. [49] indicated that high levels of antioxidant could confer resistance to HNE toxicity, and we previously reported that SC1 and EC1, metabolites with the dihydrophenyl (catechol) moiety of sesamin and episesamin, showed O_2_^−^ and •OH scavenging activity [21,28]. Hamada et al. [29] reported that SC1 and EC1 induce phase II antioxidant enzymes by activating Nrf2/ARE (antioxidant response element) signaling to reduce oxidative stress in rat pheochromocytoma PC12 cells. Additionally, Ahmad et al. [26] suppressed microglial activation and reduced infarction volume via the intraperitoneal administration sesamin in an ischemic brain stroke model. Together these results strongly suggest that sesame lignans and their metabolites might exert neuroprotective effects by directly or indirectly suppressing ROS, inhibiting the formation of RCs, and suppressing microglial activation. These results suggest that dietary factors and lifestyle changes that reduce the formation of RCs and ROS, as well as sesame lignans, could also suppress age-related decline in cognitive function.

We also found that RCs formation in the liver is increased in old SAMP10 mice compared to that in young SAMP10 mice and could be suppressed by sesame lignan intake. Other studies have indicated that sesame lignans might suppress the increase in oxidative stress in the liver. For example, Ikeda et al. [22] reported that the oral administration of 100 mg/kg sesame lignans induces glutathione peroxidase and glutathione-S-transferase in the mouse liver. In addition, sesame lignans have been reported to reduce lipid peroxide in liver injury models by inducing anti-oxidative enzymes [50]. It is increasingly becoming evident that the liver–brain axis could have a role in the development of brain inflammation, as peripheral organ inflammatory diseases including chronic inflammatory liver diseases have been associated with changes in central neural transmission [51]. We did not examine whether the liver–brain inflammation axis is involved in the cognitive decline of SAMP10 mice in this study, and further investigation is needed to characterize the influence of other organs on cognitive decline in this model system.

Cognitive decline is known to be associated with depression [52]; for example, approximately 20% of patients with dementia also experience depression [53]. Similarly, Ikeda et al. [54] reported that dementia sufferers in Japan tend to have mental and behavioral disturbances. Furthermore, a recent meta-analysis revealed that depression might be a risk factor for Alzheimer’s disease [55]. Hence, the prevention of dementia and depression might be important to maintain quality of life in the elderly. SAMP10 mice show age-related increases in immobile time in the forced swim test. As a prolonged immobile time in the forced swim test is indicative of a state of despair or depression [33], it is thought to be a useful model of senile depression. We found that sesame lignans suppressed the extension of immobile time in the forced swim test using old SAMP10 mice (Figure 3). Although this finding demonstrated that sesame lignans might exert an anti-depressant effect by inhibiting oxidative stress in the brain, the underlying mechanism is unclear and further study is needed.

The dendritic shortening and loss of spines in neurons of aged SAMP10 mice have been reported [56]. Although previous studies have shown that the change in brain weight in SAMP10 mice occurs at the age of approximately 11–12 months, under the conditions of this study, the brain weight did not change, suggesting that the loss of neurons had not yet begun. Since mice in previous studies were fed CE-2 (CLEA Japan, Inc., Tokyo, Japan), whereas mice in this study were fed a purified diet (AIN-76), it is likely that the difference in the basal diet might affect the timing of the loss of brain weight. The body weights of mice in the OS0.05 group were significantly higher than those in the OC group at 12 months of age. Since the body weight of SAMP10 mice increases until the age of seven months and then gradually decreases, sesame lignans might suppress the age-related reduction in body weight. However, survival rates in the OS groups were not higher than those in the OC group. These findings suggest that sesame lignans might extend the health span of SAMP10 mice.

## 5. Conclusions

We revealed that various RCs increase in the brains of aged mice using our comprehensive analysis method and it was suggested that sesame lignans suppress age-related cognitive decline by reducing RCs produced by oxidative stress in the brain with advancing age. Results from this study suggest that the long-term consumption of sesame lignans might be an effective way to prevent age-related decline of brain function. Our recently described method for the comprehensive analysis of RCs, which uses DH as the RC trapping agent, could also be a useful tool to estimate oxidative stress levels in the aged brain.

## Figures and Tables

**Table 1:** Body and brain weights, and survival rates of young and old mice treated with sesame lignans or vehicle.

	YC	YS0.05	OC	OS0.02	OS0.05
Body weight (g)	30.1 ± 0.3 ^ab^	28.8 ± 0.4 ^a^	30.9 ± 0.9 ^ab^	33.2 ± 1.5 ^bc^	35.0 ± 1.1 ^c^
Brain weight (mg)	467.7 ± 0.3 ^a^	467.2 ± 0.4 ^a^	469.5 ± 0.4 ^a^	465.6 ± 0.5 ^a^	468.8 ± 0.3 ^a^
Final survival rate (%)	90	85	64	75	64

Values are the mean ± SEM. Statistical analyses were performed using a one-way ANOVA followed by a Tukey–Kramer post hoc analysis. Different letters indicate significant differences (*p* < 0.05). Experimental groups are indicated as: Young control, fed basal diet (YC, *n* = 18), young, fed basal diet supplemented with 0.05% sesame lignans (YS0.05, *n* = 17), old control, fed basal diet (OC, *n* = 18), old, fed basal diet supplemented with 0.02% sesame lignans (OS0.02, *n* = 12), and old, fed diet supplemented with 0.05% sesame lignans (OS0.05, *n* = 18). One mouse in the YC group, one mouse in the OC group, and three mice in the OS0.05 group died one month after the step-through test.

## Supplementary Materials

The following are available online at https://www.mdpi.com/2072-6643/11/7/1582/s1, Figure S1: Reactive carbonyl species (RC) maps of livers obtained from SAMP10 mice in the young control, fed basal diet (YC), old control, fed basal diet (OC), old, fed basal diet supplemented with 0.02% sesame lignans (OS0.02), and old, fed diet supplemented with 0.05% sesame lignans (OS0.05). The area of each circle represents the intensity of the RCs relative to an internal standard. RC maps are presented as averages of each map (*n* = 5); Table S1: Reactive carbonyl species (RCs) in cerebral cortices; Table S2: Reactive carbonyl species (RCs) in livers.
