# Supplementary material for: Sesame Lignans Suppress Age-Related Cognitive Decline in Senescence-Accelerated Mice
Source: Nutrients. 2019 Jul 12;11(7):1582. doi: 10.3390/nu11071582 (PMC6682928; doi:10.3390/nu11071582)
Supplement: Supplementary file 1 [file nutrients-11-01582-s001.pdf]

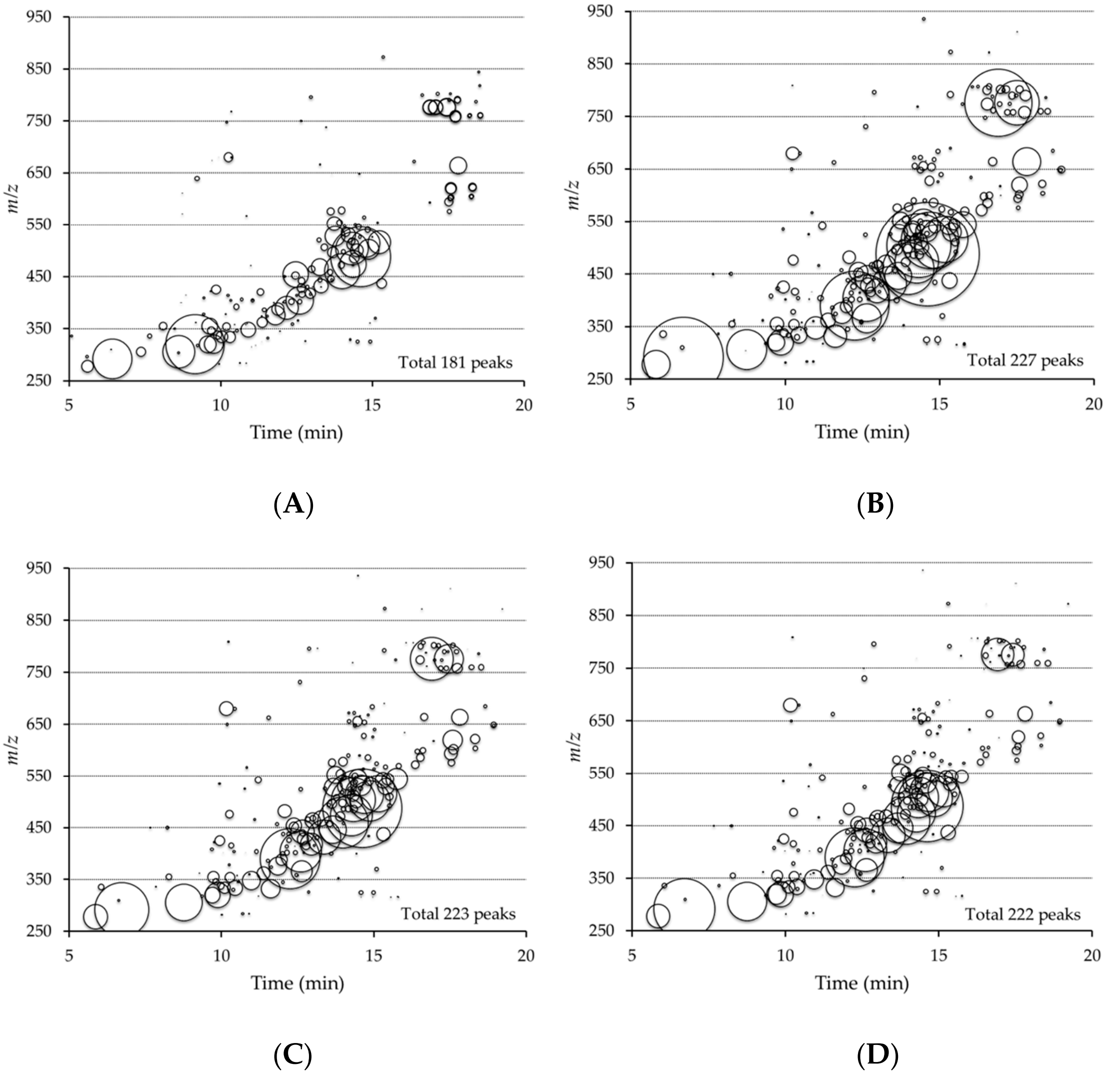

Figure S1. Reactive carbonyl species (RC) maps of livers obtained from SAMP10 mice in the (A) young control, fed basal diet (YC), (B) old control, fed basal diet (OC), (C) old, fed basal diet supplemented with 0.02% sesame lignans (OS0.02), (D) old, fed diet supplemented with 0.05% sesame lignans (OS0.05). The area of each circle represents the intensity of the RCs relative to an internal standard. RC maps are presented as averages of each map (n = 5).

Table S1. Reactive carbonyl species (RCs) in cerebral cortices

Compound	YC	OC	OS0.02	OS0.05
Formaldehyde	11768.7 ± 1493.6 [a]	19710.7 ± 4169.5 [a]	13566.0 ± 1195.7 [a]	18539.7 ± 7715.6 [a]
Acetaldehyde	536.4 ± 479.9 [a]	6817.8 ± 1297.9 [b]	3691.0 ± 341.3 [ab]	5199.9 ± 1452.5 [b]
Acrolein	767.5 ± 166.8 [a]	515.5 ± 95.9 [a]	0.0 ± 0.0 [b]	47.5 ± 47.5 [b]
Propanal	7136.7 ± 1261.8 [a]	30567.6 ± 2183.8 [b]	21438.0 ± 1069.1 [c]	25697.2 ± 2574.4 [bc]
Crotonaldehyde	29.2 ± 5.7 [a]	100.2 ± 4.5 [b]	81.3 ± 4.6 [b]	84.2 ± 6.4 [b]
Butanal	186.6 ± 160.3 [a]	10149.8 ± 688.8 [b]	3704.6 ± 546.2 [c]	4245.1 ± 1165.0 [c]
Pentanal	102.3 ± 102.3 [a]	1201.4 ± 95.4 [b]	890.2 ± 101.0 [b]	838.3 ± 137.6 [b]
2,4-Hexadienal	21.0 ± 1.6 [a]	0.0 ± 0.0 [b]	0.0 ± 0.0 [b]	0.0 ± 0.0 [b]
2-Hexenal	86.0 ± 6.5 [a]	207.1 ± 3.4 [b]	157.0 ± 6.5 [c]	168.8 ± 14.8 [c]
Hexanal	7910.9 ± 526.9 [a]	37065.0 ± 465.6 [b]	24379.0 ± 1867.8 [c]	25749.4 ± 3863.7 [c]
2,4-Heptadienal	7.1 ± 2.0 [a]	0.0 ± 0.0 [b]	0.0 ± 0.0 [b]	0.0 ± 0.0 [b]
2-Heptenal	21.0 ± 1.9 [a]	50.7 ± 2.8 [b]	38.0 ± 2.8 [c]	37.2 ± 2.2 [c]
HHE	253.1 ± 28.4 [a]	1112.3 ± 40.8 [b]	667.8 ± 24.0 [c]	783.5 ± 133.0 [c]
Heptanal	1175.9 ± 106.4 [a]	420.9 ± 61.8 [b]	300.9 ± 19.3 [b]	345.8 ± 33.9 [b]
2-Octenal	9.9 ± 0.8 [a]	20.5 ± 1.1 [a]	16.7 ± 1.4 [a]	19.4 ± 7.4 [a]
Octanal	5516.1 ± 403.0 [a]	1347.5 ± 236.5 [b]	1127.2 ± 74.3 [b]	1340.6 ± 202.9 [b]
2,4-Nonadienal	119.6 ± 5.0 [a]	0.0 ± 0.0 [b]	0.0 ± 0.0 [b]	0.0 ± 0.0 [b]
2-Nonenal	112.8 ± 3.2 [a]	113.3 ± 11.5 [a]	87.6 ± 4.4 [a]	88.6 ± 8.2 [a]
Nonanal	6211.1 ± 362.8 [a]	6783.1 ± 996.3 [a]	5201.0 ± 468.9 [a]	6529.5 ± 988.9 [a]
2,4-Decadienal	21.0 ± 2.8 [a]	174.9 ± 7.3 [b]	109.3 ± 12.9 [c]	104.4 ± 21.3 [c]
HNE	180.3 ± 20.9 [a]	429.9 ± 30.1 [b]	293.6 ± 9.2 [c]	313.9 ± 35.5 [c]
Decanal	2310.1 ± 92.8 [a]	2090.7 ± 308.3 [a]	1635.1 ± 118.3 [a]	1744.6 ± 242.3 [a]
EDE	511.5 ± 61.4 [a]	1215.6 ± 135.1 [b]	749.0 ± 53.7 [ac]	1124.5 ± 130.5 [bc]
2-Undecenal	33. 1 ± 4.0 [a]	32.6 ± 3.6 [a]	21.1 ± 1.8 [ab]	19.8 ± 2.7 [b]
Undecanal	377.2 ± 53.9 [a]	431.8 ± 96.8 [a]	280.9 ± 32.0 [a]	328.1 ± 91.2 [a]
Dodecanal	983.5 ± 183.0 [a]	1476.7 ± 243.3 [a]	1024.8 ± 145.3 [a]	1089.2 ± 226.7 [a]
Tridecanal	393.6 ± 222.5 [a]	599.2 ± 243.2 [a]	136.3 ± 117.1 [a]	296.1 ± 226.1 [a]
Pentadecanal	1282.4 ± 448.4 [a]	5823.3 ± 870.9 [b]	3997.1 ± 323.0 [b]	4421.5 ± 268.0 [b]
Hexadecanal	2521.4 ± 342.2 [a]	4763.6 ± 467.4 [b]	3831.6 ± 366.2 [ab]	4454.4 ± 438.3 [b]
8,11,14-Heptadecatrienal	225.1 ± 65.3 [a]	289.4 ± 23.5 [a]	242.3 ± 15.0 [a]	207.0 ± 28.6 [a]
8,11-Heptadecadienal	46.6 ± 8.7 [a]	133.2 ± 19.4 [b]	91.7 ± 8.5 [ab]	97.3 ± 20.3 [ab]
8-Heptadecenal	934.6 ± 67.9 [a]	2438.3 ± 188.2 [b]	2098.3 ± 69.7 [b]	1997.8 ± 152.4 [b]
Heptadecanal	201.8 ± 101.2 [a]	1729.1 ± 334.5 [b]	1177.4 ± 139.1 [b]	1280.1 ± 147.5 [b]
Octadecanal	759.0 ± 503.9 [a]	2325.3 ± 875.0 [a]	1564.7 ± 389. 5 [a]	2013.5 ± 751.4 [a]
Secosterol-A	15.5 ± 0.7 [a]	54.7 ± 2.9 [b]	39.5 ± 4.1 [c]	43.2 ± 4.4 [bc]
Secosterol-B	1.4 ± 0.8 [a]	12.3 ± 1.0 [b]	8.6 ± 3.5 [ab]	8.3 ± 2.0 [ab]

Values are the mean ± SEM (pmol/g wet tissue, n = 5). Statistical analyses were performed using a one-way ANOVA followed by a Tukey-Kramer post hoc analysis. Different letters indicate significant differences ($p < 0.05$). Experimental groups are indicated as follows: Young control, fed basal diet (YC), old control, fed basal diet (OC), old, fed basal diet supplemented with 0.02% sesame lignans (OS0.02), and old, fed diet supplemented with 0.05% sesame lignans (OS0.05). HHE: 4-hydroxy-2-hexenal, HNE: 4-hydroxy-2-nonenal, EDE: 4,5-epoxy-2-decenal.

Table S2. Reactive carbonyl species (RCs) in livers

Compound	YC	OC	OS0.02	OS0.05
Formaldehyde	10074.1 ± 4278.8 [a]	38671.6 ± 5328.9 [b]	27755.0 ± 3336.1 [b]	22204.2 ± 2914.9 [ab]
Acetaldehyde	7084.6 ± 1736.5 [a]	30945.4 ± 10660.9 [b]	15955.8 ± 2875.3 [ab]	15466.9 ± 2027.7 [ab]
Acrolein	518.0 ± 166.4 [a]	139.1 ± 101.8 [ab]	0.0 ± 0.0 [b]	0.0 ± 0.0 [b]
Glyoxal	1464.1 ± 534.1 [a]	185.7 ± 161.8 [a]	760.8 ± 237.2 [a]	393.7 ± 253.7 [a]
Propanal	13249.4 ± 2018.1 [a]	22043.3 ± 1706.8 [b]	17978.9 ± 1923.9 [ab]	18052.7 ± 1969.4 [ab]
Crotonaldehyde	151.0 ± 17.7 [a]	131.9 ± 15.0 [a]	144.5 ± 5.5 [a]	157.2 ± 17.9 [a]
Butanal	2473.6 ± 811.3 [a]	6660.9 ± 425.2 [b]	6112.1 ± 812.3 [bc]	3908.3 ± 520.9 [ac]
Pentanal	984.6 ± 170.6 [a]	2069.3 ± 134.3 [b]	1596.3 ± 141.3 [ab]	1278.6 ± 205.3 [a]
2,4-Hexadienal	29.5 ± 5.7 [a]	19.6 ± 3.2 [ab]	16.1 ± 0.5 [ab]	12.6 ± 1.6 [b]
2-Hexenal	37.6 ± 4.5 [a]	43.3 ± 6.5 [a]	35.5 ± 1.8 [a]	34.3 ± 4.0 [a]
Hexanal	3394.8 ± 383.0 [a]	7302.2 ± 363.4 [b]	4457.4 ± 228.4 [a]	4534.3 ± 508.8 [a]
2,4-Heptadienal	6.7 ± 2.4 [a]	7.9 ± 2.2 [a]	7.1 ± 1.3 [a]	4.4 ± 1.4 [a]
2-Heptenal	31.0 ± 7.3 [a]	65.3 ± 7.6 [b]	46.9 ± 3.5 [ab]	40.1 ± 3.6 [a]
HHE	31.8 ± 3.6 [a]	1839.6 ± 434.5 [b]	774.6 ± 149.9 [a]	743.2 ± 232.9 [a]
Heptanal	735.3 ± 125.3 [a]	650.5 ± 37.7 [ab]	549.8 ± 35.8 [ab]	429.2 ± 48.9 [b]
2-Octenal	16.0 ± 1.0 [a]	32.4 ± 6.5 [b]	18.3 ± 2.6 [ab]	12.6 ± 2.3 [a]
Octanal	3327.6 ± 635.0 [a]	2456.2 ± 247.9 [ab]	2100.7 ± 178.9 [ab]	1545.3 ± 193.4 [b]
2,4-Nonadienal	66.0 ± 10.6 [a]	1.0 ± 1.0 [b]	0.0 ± 0.0 [b]	1.6 ± 1.6 [b]
2-Nonenal	252.4 ± 24.0 [ab]	280.9 ± 13.2 [b]	254.5 ± 9.8 [b]	192.7 ± 9.3 [a]
Nonanal	5550.6 ± 983.4 [a]	10063.4 ± 920.2 [b]	8261.5 ± 555.3 [ab]	6288.1 ± 390.3 [a]
2,4-Decadienal	32.3 ± 2.8 [a]	105.3 ± 9.7 [b]	66.6 ± 10.1 [c]	64.3 ± 6.3 [c]
HNE	70.2 ± 1.5 [a]	111.0 ± 9.1 [b]	62.8 ± 5.6 [a]	69.4 ± 5.9 [a]
Decanal	1782.2 ± 351.3 [a]	3033.8 ± 233.5 [b]	2846.4 ± 219.0 [ab]	2143.2 ± 290.0 [ab]
EDE	165.0 ± 16.7 [ab]	328.2 ± 76.5 [a]	79.7 ± 37.6 [b]	81.4 ± 24.0 [b]
2-Undecenal	53.1 ± 7.4 [a]	49.6 ± 2.4 [a]	48.2 ± 1.9 [a]	41.7 ± 1.8 [a]
Undecanal	495.8 ± 115.8 [a]	1108.6 ± 83.8 [b]	1097.6 ± 99.0 [b]	815.3 ± 112.8 [ab]
Dodecanal	1130.5 ± 319.4 [a]	2797.2 ± 186.7 [b]	3000.9 ± 298.5 [b]	2382.2 ± 374.4 [b]
Tridecanal	715.2 ± 387.4 [a]	1911.7 ± 196.3 [ab]	2128.0 ± 234.0 [b]	1749.6 ± 329.1 [ab]
Tetradecanal	47.8 ± 46.8 [a]	659.4 ± 100.7 [b]	654.7 ± 68.5 [b]	415.3 ± 131.7 [ab]
Pentadecanal	1205.4 ± 403.0 [a]	5238.0 ± 467.9 [b]	4706.6 ± 430.1 [b]	3599.4 ± 513.2 [b]
Hexadecanal	2373.4 ± 423.5 [a]	10276.1 ± 730.9 [b]	5972.9 ± 389.8 [c]	5048.0 ± 744.5 [c]
8,11,14-Heptadecatrienal	311.1 ± 53.6 [a]	488.4 ± 125.8 [a]	288.0 ± 66.7 [a]	180.3 ± 46.8 [a]
8,11-Heptadecadienal	65.6 ± 20.2 [a]	307.4 ± 26.5 [b]	256.5 ± 10.4 [b]	239.5 ± 19.2 [b]
8-Heptadecenal	221.1 ± 35.8 [a]	260.4 ± 16.4 [a]	206.1 ± 13.0 [a]	232.9 ± 21.3 [a]
Heptadecanal	104.7 ± 71.8 [a]	1209.6 ± 122.8 [b]	1006.9 ± 68.3 [bc]	691.3 ± 148.4 [c]
Octadecanal	406.4 ± 191.9 [a]	2699.1 ± 268.9 [b]	1727.3 ± 182.3 [c]	1071.2 ± 239.6 [ac]
Secosterol-A	12.0 ± 4.1 [a]	105.2 ± 28.4 [b]	47.6 ± 12.0 [ab]	36.8 ± 14.7 [ab]
Secosterol-B	0.5 ± 0.4 [a]	31.4 ± 10.7 [b]	12.9 ± 5.4 [ab]	8.1 ± 3.8 [ab]

Values are the mean ± SEM (pmol/g wet tissue, n = 5). Statistical analyses were performed using a one-way ANOVA followed by a Tukey-Kramer post hoc analysis. Different letters indicate significant differences ($p < 0.05$). Experimental groups are indicated as follows: Young control, fed basal diet (YC), old control, fed basal diet (OC), old, fed basal diet supplemented with 0.02% sesame lignans (OS0.02), and old, fed diet supplemented with 0.05% sesame lignans (OS0.05). HHE: 4-hydroxy-2-hexenal, HNE: 4-hydroxy-2-nonenal, EDE: 4,5-epoxy-2-decenal.
